# Association between pregnancy cumulative glycemic exposure and postpartum glucose metabolism outcomes at 4–7 years: a cohort study

**DOI:** 10.3389/fmed.2026.1876136

**Published:** 2026-08-05

**Authors:** Dan Zhao, Ning Yuan, Jianbin Sun, Xiaomei Zhang, Linong Ji

**Affiliations:** 1 Department of Endocrinology, Peking University People’s Hospital, Beijing, China; 2 Department of Endocrinology, Peking University International Hospital, Beijing, China

**Keywords:** continuous glucose monitoring, cumulative glycemic exposure, gestational diabetes mellitus, glycemic metabolism abnormality, postpartum, predictor

## Abstract

**Objective:**

This study aimed to investigate the association between cumulative glycemic exposure (CGE) during pregnancy and glycemic metabolism abnormality (GMA) at 4–7 years postpartum and to compare its predictive value with that of a single fasting blood glucose (FBG) measurement.

**Methods:**

In this retrospective–prospective cohort study, 120 pregnant women were included from an existing cohort. CGE was calculated using the trapezoidal rule, and subjects were categorized into tertiles. A logistic regression analysis evaluated the association between CGE and GMA, and restricted cubic splines were used to evaluate dose–response relationships. The interaction between CGE and pre-pregnancy body mass index (BMI) was examined. Predictive performance was compared using receiver operating characteristic (ROC) curves.

**Results:**

Higher CGE was associated with an increased incidence of GMA [tertile 1 (T1): 10.0%, T2: 35.0%, T3: 65.0%]. Each 1-unit increment in CGE independently increased GMA risk (odds ratio = 1.040, 95% confidence interval: 1.01–1.07, *p* = 0.028). Compared with T1, the risk of GMA was 3.85-fold higher in T2 and 15.82-fold higher in T3 (*p* < 0.05). FBG at 8 and 24 weeks, but not 34 weeks, significantly predicted GMA. A non-linear dose–response between CGE and GMA was observed (inflection point: 192.67 mmol/L·week). An additive interaction between CGE and pre-pregnancy BMI was observed. The combined model (CGE + pre-pregnancy BMI) provided the best prediction [area under the curve (AUC): 0.843, sensitivity: 85.5%, specificity: 79.1%].

**Conclusion:**

CGE during pregnancy is an independent predictor of postpartum GMA. In clinical practice, adding pre-pregnancy BMI improves risk stratification, supporting the early identification of high-risk women.

**Clinical trial registration:**

Identifier ChiCTR2300067592.

## 1 Introduction

Gestational diabetes mellitus (GDM) is defined as hyperglycemia first detected or recognized during pregnancy (1, 2). Its increasing global prevalence poses long-term metabolic risks for both mothers and offspring (3, 4). Women with GDM have a 7- to 10-fold increased risk of type 2 diabetes (T2DM) postpartum; however, glycemic abnormalities often manifest years after delivery (5–7). The majority of existing studies have relied on single fasting blood glucose (FBG) measurements or oral glucose tolerance testing (OGTT), which may not adequately capture the sustained impact of cumulative hyperglycemic exposure during pregnancy on long-term metabolic outcomes. More recently, cumulative exposure metrics, such as the area under the curve (AUC), have been applied to evaluate the contributions of long-term predictors, such as blood pressure and glycemia, to cardiovascular and cerebrovascular outcomes (8, 9), which remain underexplored in postpartum GDM cohorts.

Therefore, leveraging data from a previously established GDM cohort, this study aimed to quantify cumulative glycemic exposure (CGE) during pregnancy and investigate its association with glycemic metabolism abnormality (GMA) at 4–7 years postpartum. In addition, we aimed to compare the predictive utility of CGE with that of single FBG measurements obtained at different trimesters and to explore the potential effect modification by pre-pregnancy body mass index (BMI), thereby providing a scientific foundation for the long-term risk stratification and management of women with prior GDM.

## 2 Materials and methods

### 2.1 Participants

**Figure 1:**
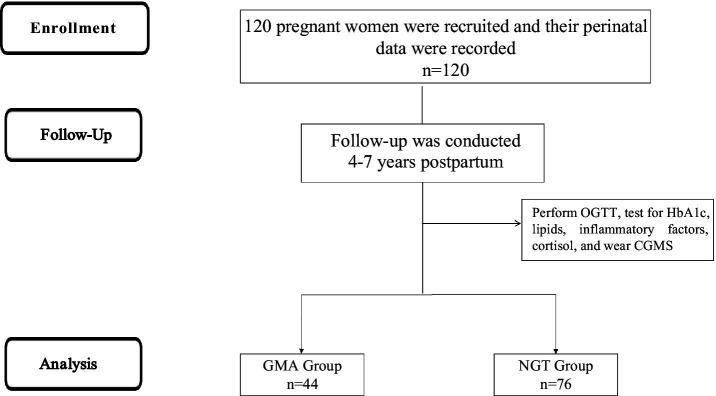
Flow diagram. CGMS, continuous glucose monitoring systems; GMA, glucose metabolism anomaly; HbA1c, glycosylated hemoglobin; NGT, normal glycemic tolerance; OGTT, oral glucose tolerance tests.

This study was a secondary analysis based on a previously established GDM cohort (10). The original cohort was a maternal cohort recruited at Peking University International Hospital between October 2017 and August 2019 and consisted of a total of 120 pregnant women, including 60 with a confirmed diagnosis of GDM and 60 normoglycemic controls. At 4–7 years postpartum, all participants returned to the hospital for a general physical examination, biochemical testing, a 75-g oral glucose tolerance test, and 7-day retrospective continuous glucose monitoring (CGM). Participants were eligible if they met the following criteria: (1) age ≥18 years; (2) singleton pregnancy; (3) complete perinatal clinical data; and (4) voluntary participation with written informed consent. Participants were excluded if they met the following criteria: (1) pre-pregnancy diabetes mellitus; (2) multiple gestation; (3) autoimmune disease; (4) severe hepatic or renal insufficiency; (5) long-term use of antidepressants or glucocorticoids; and (6) use of glucose-lowering agents or insulin during follow-up. The research followed the guidelines of the Strengthening the Reporting of Observational Studies in Epidemiology (STROBE) statement (Figure 1).

### 2.2 Data collection

Detailed descriptions of data collection have been previously published (10, 11). In brief, perinatal information was obtained from the electronic medical record system at the time of cohort establishment. At the 4–7 year postpartum follow-up, anthropometric parameters, including blood pressure, height, weight, body fat percentage, and waist and hip circumferences, were measured using standardized protocols and calibrated equipment. All participants underwent 7-day retrospective continuous glucose monitoring (iPro2; Medtronic, Northridge, CA, USA) with twice-daily capillary glucose calibration. Fasting venous blood samples were collected for the assessment of lipid profiles, high-sensitivity C-reactive protein (CRP), cortisol, glycated hemoglobin (HbA1c), and a standard 75-g OGTT with insulin measurements. All biochemical assays were performed in the certified laboratory of Peking University International Hospital under strict internal and external quality control procedures.

### 2.3 Definitions and calculations

CGE was assessed by calculating the AUC for FBG during pregnancy using the trapezoidal rule, as follows: CGE= $\sum (\frac{G_{i}+G_{i+1}}{2}\times \mathrm{\Delta t})$ , where *Gi* represents the FBG value at the *i*-th measurement and *Δt* denotes the interval, in weeks, between consecutive measurements. Fasting venous blood samples were collected in the first trimester (8 ± 2 weeks), second trimester (24 ± 2 weeks), and third trimester (34 ± 2 weeks), and FBG concentrations were determined using the hexokinase method. FBG values from 34 weeks to delivery were imputed using the last observation carried forward approach. Participants were categorized into tertiles (T1, lowest; T2 and T3, highest) based on the calculated CGE.

The primary outcome was GMA at 4–7 years postpartum, defined as meeting any of the following criteria: (1) impaired fasting glucose (IFG): fasting plasma glucose of 6.1–6.9 mmol/L; (2) impaired glucose tolerance (IGT): 2-h plasma glucose of 7.8–11.0 mmol/L following a 75-g oral glucose load; or (3) T2DM: fasting plasma glucose of ≥7.0 mmol/L or 2-h plasma glucose of ≥11.1 mmol/L. The diagnosis was based on the Chinese guidelines for the prevention and treatment of diabetes and the 1999 World Health Organization (WHO) criteria (12).

The diagnostic criteria for GDM in this study were based on the criteria of the International Association of Diabetes and Pregnancy Study Groups (13). BMI was calculated as follows: $\;\frac{\text{Weight}(\mathrm{kg})}{{\text{Height}}^{2(}m^{2)}}$ . Homeostasis model assessment for insulin resistance (HOMA-IR) was calculated as follows: $\frac{\mathrm{FBG}\times \text{FINS}}{405}$ ; Homeostasis model assessment for β-cell function (HOMA-β) was calculated as follows: $\frac{20\times \text{FINS}}{\mathrm{FBG}-3.5}$ . In the above formula, blood glucose units are mg/dL, and insulin units are μU/mL (to convert to pmol/L, multiply by 6.945).

### 2.4 Statistical analysis

Statistical analyses were performed using SPSS 29.0 software. Data normality was assessed using the Kolmogorov–Smirnov test. Normally distributed continuous variables were expressed as *mean ± standard deviation (x̄ ± SD)*, non-normally distributed continuous variables were expressed as *median (interquartile range)*, and categorical variables were expressed as absolute numbers and percentages.

Group differences were assessed using an analysis of variance (ANOVA), the Kruskal–Wallis, *χ*^2^ test, or Fisher’s exact tests, as appropriate. A binary logistic regression analysis was performed to evaluate the associations between CGE and individual FBG measurements with GMA, with results presented as odds ratios (OR) and 95% confidence intervals (CI). *Post-hoc* multiple comparisons were performed using the Bonferroni correction when the *p*-value was <0.05. Non-linear relationships were explored using restricted cubic spline (RCS) analysis with four knots. Subgroup analyses were performed by age and pre-pregnancy BMI, with the interaction being tested. Predictive performance was evaluated using the receiver operating characteristic (ROC) curves; AUC, sensitivity, specificity, and optimal thresholds (Youden index) were computed. A two-sided *p*-value of < 0.05 was considered statistically significant.

Given the limited number of events (*n* = 44), we applied the “one in ten” rule (events per variable >10) when constructing logistic regression models. Candidate covariates were selected based on clinical plausibility and univariate associations (*p* < 0.10), with a maximum of four covariates allowed in the final multivariable model. Multicollinearity was assessed using variance inflation factors (VIFs), with a threshold of >5 indicating significant collinearity. To avoid collinearity, CGE and FBG levels were not entered together in any regression model, since CGE was derived from serial FBG measurements. Internal validation of the ROC models was performed using bootstrap resampling (1,000 iterations) to obtain optimism-corrected AUC estimates.

## 3 Results

### 3.1 Baseline characteristics according to CGE tertiles

A total of 120 participants were included in this study and categorized into tertile groups based on CGE levels: T1 (lowest, *n* = 40), T2 (middle, *n* = 40), and T3 (highest, *n* = 40). As shown in Table 1, the mean gestational age at delivery was 38.34 weeks. CGE values differed significantly across the three groups (*p* < 0.001). Correspondingly, FBG levels at 8 weeks, 24 weeks, and 34 weeks of gestation increased progressively with higher CGE (all *p* < 0.001). Significant differences among the groups were also observed for maternal age (*p* = 0.024) and pre-pregnancy BMI (*p* = 0.042). The proportion of GDM increased progressively from T1 to T3 (*p* < 0.001). No statistically significant differences were found among the three groups for gestational week, gestational weight gain (GWG), parity, blood pressure, lipid profiles, or C-reactive protein (all *p* > 0.05).

**Table 1:** Early-pregnancy baseline clinical characteristics of pregnant women according to the tertiles of CGE levels.

Characteristics		Total (*N* = 120)		Tertile 1 (*N* = 40)		Tertile 2 (*N* = 40)		Tertile 3 (*N* = 40)		*F/H/χ* ^2^	*p*	Comparison between the groups
CGE*		194.45 ± 24.44		176.18 ± 6.92		192.05 ± 3.57		215.13 ± 31.21		44.46	<0.001	1 < 2 < 3
FBG—8 weeks*	mmol/L	4.98 ± 0.52		4.65 ± 0.29		4.89 ± 0.27		5.40 ± 0.59		29.35	<0.001	1 < 2 < 3
FGB—24 weeks*	mmol/L	4.82 ± 0.73		4.22 ± 0.27		4.75 ± 0.19		5.48 ± 0.82		67.82	<0.001	1 < 2 < 3
FBG—34 weeks*	mmol/L	4.73 ± 1.00		4.25 ± 0.39		4.72 ± 0.26		5.21 ± 1.52		11.72	<0.001	1 < 3
Age – pregnancy*	years	31.51 ± 3.61		30.90 ± 3.30		30.85 ± 3.77		32.78 ± 3.49		4.96	0.024	2 < 3
BMI–pre-pregnancy*	kg/m^2^	22.99 ± 3.08		22.01 ± 2.67		23.43 ± 3.49		23.55 ± 2.86		2.62	0.042	1 < 3
Gestational week		38.34 ± 1.82		38.50 ± 1.63		38.17 ± 2.46		38.35 ± 1.66		0.31	0.731	
GWG	kg	12.39 ± 4.39		13.47 ± 4.91		11.77 ± 3.46		11.92 ± 4.58		2.10	0.126	
Parity (prior deliveries)	≥1	54	45.0%	15	37.5%	21	52.5%	18	45.0%	2.62	0.403	
SBP	mmHg	109.90 ± 11.77		109.75 ± 10.59		109.22 ± 9.93		110.72 ± 14.53		0.16	0.848	
DBP	mmHg	67.90 ± 12.90		66.05 ± 8.05		66.52 ± 8.62		71.12 ± 18.78		1.91	0.152	
TC	mmol/L	3.95 ± 0.86		3.91 ± 1.06		3.94 ± 0.56		4.00 ± 0.89		0.11	0.889	
TG	mmol/L	1.12 ± 0.77		0.91 ± 0.55		1.17 ± 0.63		1.29 ± 0.73		1.99	0.082	
HDL-C	mmol/L	1.92 ± 0.81		1.51 ± 0.26		1.37 ± 0.31		2.19 ± 0.72		0.84	0.431	
LDL-C	mmol/L	2.34 ± 0.82		2.16 ± 0.70		2.61 ± 0.32		2.25 ± 0.62		1.47	0.233	
CRP	mg/L	1.98 (0.95, 4.40)		1.97 (0.92, 3.30)		1.61 (0.63, 6.17)		2.40 (1.02, 4.48)		1.88	0.097	
GDM*		60	50%	7	17.5%	15	37.5%	38	95%	47.65	<0.001	1 < 3; 2 < 3

BMI, body mass index; CGE, cumulative glycemic exposure; CPR, C-reactive protein; DBP, diastolic blood pressure; FBG, fasting blood glucose; GDM, gestational diabetes mellitus; GWG, gestational weight gain; HDL-C, high-density lipoprotein cholesterol; LDL-L, low-density lipoprotein cholesterol; SBP, systolic blood pressure; TC, total cholesterol; TG, triglycerides. *p<0.05.

### 3.2 Postpartum metabolic outcomes according to CGE tertiles

Table 2 presents the clinical characteristics of the study participants at 4–7 years postpartum. The duration of postpartum follow-up (i.e., offspring age) and postpartum BMI did not differ significantly among the groups. Systolic blood pressure (SBP) was comparable across the groups, whereas diastolic blood pressure (DBP) increased progressively with higher CGE (*p* = 0.039). The prevalence of GMA increased significantly with increasing CGE (*p* = 0.022). Consistently, FBG (*p* = 0.010) and postprandial blood glucose (PBG, *p* < 0.001) exhibited stepwise elevations across the tertiles. Fasting insulin levels were also higher in the upper CGE tertiles (*p* = 0.050). The HOMA-IR increased in parallel with CGE (*p* = 0.047), whereas HOMA-*β* did not differ significantly among the groups (*p* = 0.847). In terms of glycemic variability, higher CGE was associated with elevated mean blood glucose (MBG, *p* < 0.001), maximum blood glucose (Max BG, p < 0.001), SD (p < 0.001), coefficient of variation (CV, *p* = 0.004), mean amplitude of glycemic excursion (MAGE, p < 0.001), large amplitude of glycemic excursion (LAGE, *p* = 0.003), mean of daily differences (MODDs, p < 0.001), and average daily risk range (ADRR, p < 0.001). Time in range (TIR) did not differ significantly among the groups (*p* = 0.337). In addition, triglyceride (TG) concentrations varied significantly across the three groups (*p* = 0.046), whereas total cholesterol (TC), high-density lipoprotein cholesterol (HDL-C), and low-density lipoprotein cholesterol (LDL-C) did not differ significantly among the groups.

**Table 2:** Clinical characteristics of patients at 4–7 years postpartum grouped according to the tertiles of CGE levels.

Characteristic		Total (*N* = 120)		Tertile 1 (*N* = 40)		Tertile 2 (*N* = 40)		Tertile 3 (*N* = 40)		*F/χ* ^2^	*p*	Comparison between the groups
Age—offspring	years	5.84 ± 0.98		5.92 ± 1.16		5.80 ± 0.91		5.80 ± 0.88		0.27	0.764	
BMI	kg/m^2^	23.28 ± 3.70		22.68 ± 3.29		23.44 ± 4.02		23.72 ± 3.78		0.84	0.433	
SBP	mmHg	111.57 ± 13.20		109.52 ± 8.24		112.25 ± 15.72		112.95 ± 14.48		0.74	0.475	1 < 3
DBP*	mmHg	70.19 ± 10.89		68.27 ± 7.08		69.50 ± 8.67		72.80 ± 12.08		2.42	0.039	
GMA*		44	36.7%	4	10.0%	14	35.0%	26	65%	14.73	0.022	1 < 2 < 3
	IFG	4	3.3%	0	0.0%	1	2.5%	3	7.5%			
	IGT	27	22.5%	2	5.0%	10	25.0%	15	37.5%			
	T2DM	13	10.8%	2	5.0%	3	7.5%	8	20.0%			
FBG*	mmol/L	5.53 ± 1.54		5.18 ± 1.37		5.29 ± 0.79		6.13 ± 2.02		4.76	0.010	1 < 3; 2 < 3
PBG*	mmol/L	7.21 ± 2.89		5.87 ± 1.08		7.01 ± 1.97		8.76 ± 4.01		4.68	<0.001	1 < 3; 2 < 3
Insulin*		12.15 ± 8.89		9.54 ± 5.61		12.59 ± 9.92		14.31 ± 9.98		3.04	0.050	1 < 3
HOMA-β		135.31 ± 78.46		134.81 ± 64.55		140.62 ± 91.52		130.68 ± 78.66		0.16	0.847	
HOMA-IR*		3.18 ± 1.85		2.41 ± 1.62		3.12 ± 1.56		4.01 ± 1.69		2.66	0.047	1 < 3
MBG*	mmol/L	6.82 ± 2.64		5.39 ± 1.79		6.60 ± 2.10		8.46 ± 2.95		12.89	<0.001	1 < 3; 2 < 3
Max BG*	mmol/L	9.47 ± 2.67		8.14 ± 1.32		9.40 ± 2.40		10.87 ± 3.22		7.86	<0.001	1 < 3; 2 < 3
Min BG	mmol/L	3.61 ± 1.13		3.52 ± 0.88		3.57 ± 0.91		3.73 ± 1.50		0.35	0.700	
SD*	mmol/L	0.92 ± 0.45		0.72 ± 0.21		0.88 ± 0.42		1.17 ± 0.54		9.35	<0.001	1 < 3; 2 < 3
CV*	%	16.28 ± 6.46		13.99 ± 4.69		15.37 ± 6.10		19.48 ± 7.18		5.79	0.004	1 < 3; 2 < 3
MAGE*	mmol/L	2.12 ± 1.04		1.63 ± 0.51		1.99 ± 0.68		2.73 ± 1.39		10.47	<0.001	1 < 3; 2 < 3
LAGE*	mmol/L	5.86 ± 2.70		4.61 ± 1.71		5.78 ± 2.71		7.18 ± 2.92		6.17	0.003	1 < 3; 2 < 3
MODD*	mmol/L	0.82 ± 0.34		0.64 ± 0.19		0.77 ± 0.25		1.04 ± 0.43		13.35	<0.001	1 < 3; 2 < 3
ADRR*	mmol/L	0.62 ± 0.37		0.29 ± 0.20		0.64 ± 0.39		0.94 ± 0.15		76.85	<0.001	1 < 2 < 3
TC	mmol/L	4.72 ± 0.83		4.62 ± 0.81		4.61 ± 0.82		4.94 ± 0.84		2.10	0.126	
TG*	mmol/L	1.27 ± 1.18		1.06 ± 1.02		1.09 ± 0.83		1.65 ± 0.52		3.16	0.046	1 < 3
HDL-C	mmol/L	1.36 ± 0.29		1.37 ± 0.27		1.36 ± 0.27		1.35 ± 0.33		0.02	0.976	
LDL-C	mmol/L	2.89 ± 0.71		2.84 ± 0.61		2.84 ± 0.75		3.00 ± 0.76		0.68	0.508	

ADRR, average daily risk range; BMI, body mass index; CV, coefficient of variation; DBP, diastolic blood pressure; FBG, fasting blood glucose; GMA, glucose metabolism anomaly; HDL-C, high-density lipoprotein-cholesterol; HOMA-IR, homeostasis model assessment for insulin resistance; HOMA-β, homeostasis model assessment for β-cell function; IFG, impaired fasting glucose; IGT, impaired glucose tolerance; LAGE, largest amplitude of glycemic excursions; LDL-L, low-density lipoprotein cholesterol; MAGE, mean amplitude of glycemic excursion; Max BG, maximum blood glucose; MBG, mean blood glucose; Min BG, minimum blood glucose; MODD, mean of daily difference; PBG, postprandial blood glucose; SBP, systolic blood pressure; SD, standard deviation; TC, total cholesterol; TG, triglycerides; TIR, time in target range; T2DM, type 2 diabetes mellitus. *p<0.05.

### 3.3 Association of CGE and FBG with GMA

Table 3 presents the associations between CGE and trimester-specific FBG measurements with GMA at 4–7 years postpartum. In the unadjusted model (Model 1), each 1-unit increase in CGE was associated with a 5% higher risk of GMA (OR = 1.05, 95% CI: 1.02–1.08, *p* = 0.003). This association remained significant after adjusting for gestational weight gain, parity, gestational age at delivery, and offspring age (Model 2) (adjusted OR (aOR) = 1.042, 95% CI: 1.01–1.07, p = 0.003). Compared with the T1 group, participants in the T2 and T3 groups exhibited significantly elevated risks of GMA (T2: aOR = 4.85, 95% CI: 1.43–16.42, *p* = 0.011; T3: aOR = 16.71, 95% CI: 4.93–56.63, *p* < 0.001). Among trimester-specific FBG measurements, FBG levels at 8 weeks of gestation (aOR = 10.99, 95% CI: 3.49–34.69, p < 0.001) and at 24 weeks of gestation (aOR = 2.62, 95% CI: 1.30–5.26, *p* = 0.007) were significant predictors of GMA, whereas FBG at 34 weeks of gestation was not significantly associated with GMA (aOR = 1.30, 95% CI: 0.81–2.09, *p* = 0.276). To further assess the robustness of the association, we constructed an additional model (Model 3) with further adjustment for pre-pregnancy BMI. The results remained essentially unchanged: Each 1-unit increment in CGE was associated with a 4.0% higher risk of GMA (aOR = 1.040, 95% CI: 1.01–1.07, *p* = 0.028), and the risk for T2 and T3 versus T1 remained significantly elevated (T2: aOR = 3.85, 95% CI: 1.43–16.42, *p* = 0.049; T3: aOR = 15.82, 95% CI: 4.51–55.48, *p* < 0.001). CGE and FBG levels were analyzed using separate models to avoid collinearity, given their mathematical interdependence.

**Table 3:** Logistic regression analysis of the associations between CGE and FBG with GMA.

Metric category	Model 1		Model 2		Model 3	
	OR (95% CI)	*p*	aOR (95%CI)	*p*	aOR (95% CI)	*p*
CGE	1.05 (1.02, 1.08)	0.003	1.042 (1.01, 1.07)	0.003	1.040 (1.01, 1.07)	0.028
Tertile 1	Ref		Ref		Ref	
Tertile 2	5.18 (1.43, 18.79)	0.012	4.85 (1.43, 16.42)	0.011	3.85 (0.94, 15.64)	0.049
Tertile 3	24.22 (6.23, 94.11)	<0.001	16.71 (4.93, 56.63)	<0.001	15.82 (4.51, 55.48)	<0.001
FBG-8	13.78 (4.05, 46.87)	<0.001	10.99 (3.49, 34.69)	<0.001	10.36 (2.74, 29.64)	<0.001
FBG-24	2.76 (1.27, 6.02)	0.011	2.62 (1.30, 5.26)	0.007	2.03 (1.29, 4.16)	0.035
FBG-34	1.24 (0.80, 1.93)	0.333	1.30 (0.81, 2.09)	0.276	1.18 (0.78, 1.78)	0.427

Model 1: unadjusted.

Model 2: adjusted for weight-add, parity, gestational weeks, and age-offspring.

Model 3: adjusted for weight-add, parity, gestational weeks, age-offspring, and pre-pregnancy body mass index.

CGE, cumulative glycemic exposure; CI, confidence interval; FBG, fasting blood glucose; GMA, glucose metabolism anomaly; OR, odds ratio.

**Figure 2:**
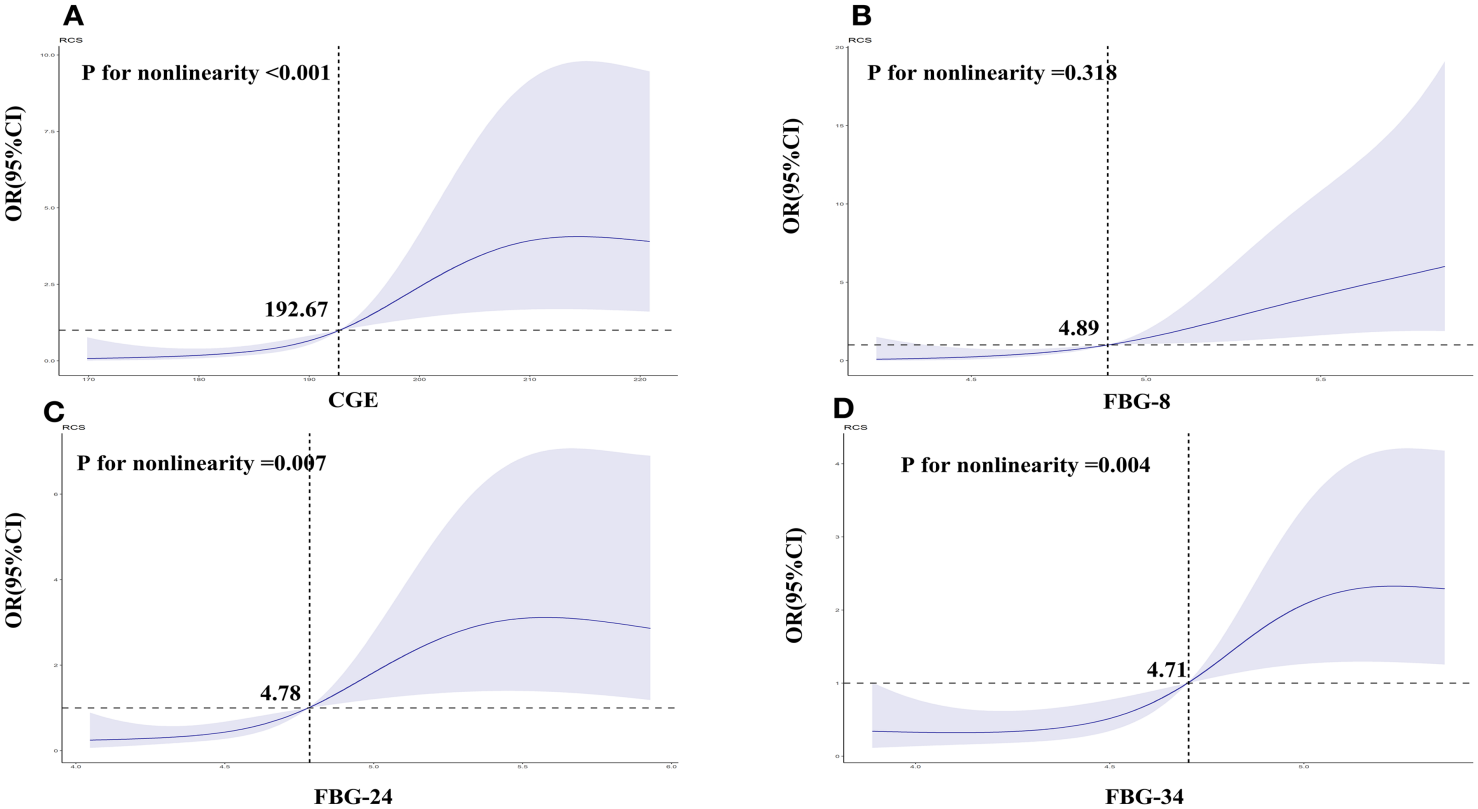
Restricted cubic spline curves of the association between CGE and FBG with GMA. **(A)** CGE (P for nonlinearity < 0.001). **(B)** FBG at 8 weeks (*P* = 0.316). **(C)** FBG at 24 weeks (*P* = 0.007). **(D)** FBG at 34 weeks (*P* = 0.004). Odds ratios are indicated by solid red lines and 95% CIs are indicated by shaded areas. Abbreviation: CGE, Cumulative glycemic exposure; CI, confidence interval; FBG, fasting blood glucose; GMA, glucose metabolism anomaly; OR, odds ratio.

We subsequently used RCS to examine potential non-linear dose–response relationships between CGE, trimester-specific FBG, and GMA. A non-linear association was observed between CGE and GMA. As illustrated in Figure 2A, the risk of postpartum GMA remained relatively stable when CGE was below 192.67 mmol/L·week, whereas it increased progressively with higher CGE values at or above this threshold (*p* < 0.001). The associations between FBG at 24 weeks (Figure 2C, *p* = 0.007) and 34 weeks (Figure 2D, *p* = 0.004) of gestation and GMA also exhibited non-linear patterns, with respective inflection points of 4.78 mmol/L and 4.71 mmol/L. In contrast, FBG at 8 weeks of gestation exhibited a linear relationship with GMA (Figure 2B, *p* = 0.318).

### 3.4 Subgroup and interaction analyses

**Figure 3:**
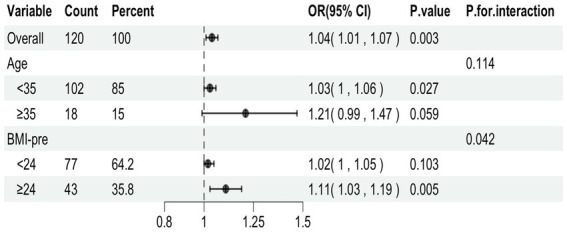
Subgroup analyses for GMA. BMI, body mass index; CI, confidence interval; GMA, glucose metabolism anomaly; OR, odds ratio.

We subsequently performed subgroup and interaction analyses stratified by age and pre-pregnancy BMI (Figure 3). In the analysis stratified by age, no significant interaction was observed between the effect estimates of CGE in the <35 years group (*n* = 102, 85%) and the ≥35 years group (n = 18, 15%) (p for interaction = 0.114). When stratified by pre-pregnancy BMI, the association between CGE and GMA did not reach statistical significance in the subgroup with BMI < 24 kg/m^2^ (n = 77, 64.2%; OR = 1.02, 95% CI: 1.00–1.05, *p* = 0.103), whereas it was significantly stronger in the subgroup with a BMI of ≥ 24 kg/m^2^ (n = 43, 35.8%; OR = 1.11, 95% CI: 1.03–1.19, *p* = 0.005). The *p*-value for the interaction between the two subgroups was 0.042, indicating that the detrimental effect of elevated CGE on long-term GMA is more pronounced among women with pre-pregnancy overweight or obesity.

Table 4 further shows the interaction between CGE and pre-pregnancy BMI on the risk of GMA. The multiplicative interaction term (CGE × pre-pregnancy BMI) was not statistically significant (OR = 1.01, 95% CI: 0.99–1.02, *p* = 0.128). An additive interaction analysis revealed a relative excess risk due to interaction (RERI) of 1.02 (95% CI: 0.50–1.52), with the confidence interval excluding zero; the attributable proportion due to interaction (AP) was 15.3% (95% CI: 12–18%); and the synergy index (S) was 1.21 (95% CI: 1.16–1.27). These findings indicate that the combined effect exceeds the sum of individual effects by 1.02 units, with the interaction accounting for 15.3% of the total risk, thereby suggesting a positive additive interaction. However, given the limited sample size in the BMI ≥ 24 kg/m^2^ subgroup (*n* = 43) and the non-significant multiplicative interaction, these additive interaction findings should be interpreted as exploratory and require confirmation in larger studies.

**Table 4:** Multiplicative interaction and additive interaction between the CGE and BMI-pre-pregnancy in GMA.

Metric category	Multiplicative interaction					Additive interaction		
	*β*	SE.	Wald *χ*^2^	OR (95% CI)	*p*		Estimated value	95% CI
CGE	0.195	0.023	1.67	0.82 (0.61, 1.01)	0.196	RERI	1.02	0.50, 1.52
BMI-pre	1.691	0.256	1.75	0.18 (0.15, 2.25)	0.185	AP (%)	15.3%	0.12, 0.18
CGE and BMI-pre	0.010	0.036	2.31	1.01 (0.99, 1.02)	0.128	S	1.21	1.16, 1.27

AP, attributable proportion due to interaction; BMI, body mass index; CGE, cumulative glycemic exposure; CI, confidence interval; GMA, glucose metabolism anomaly; OR, odds ratio; RERI, relative excess risk due to interaction; S, the synergy index.

### 3.5 Predictive performance of CGE and FBG for GMA

**Figure 4:**
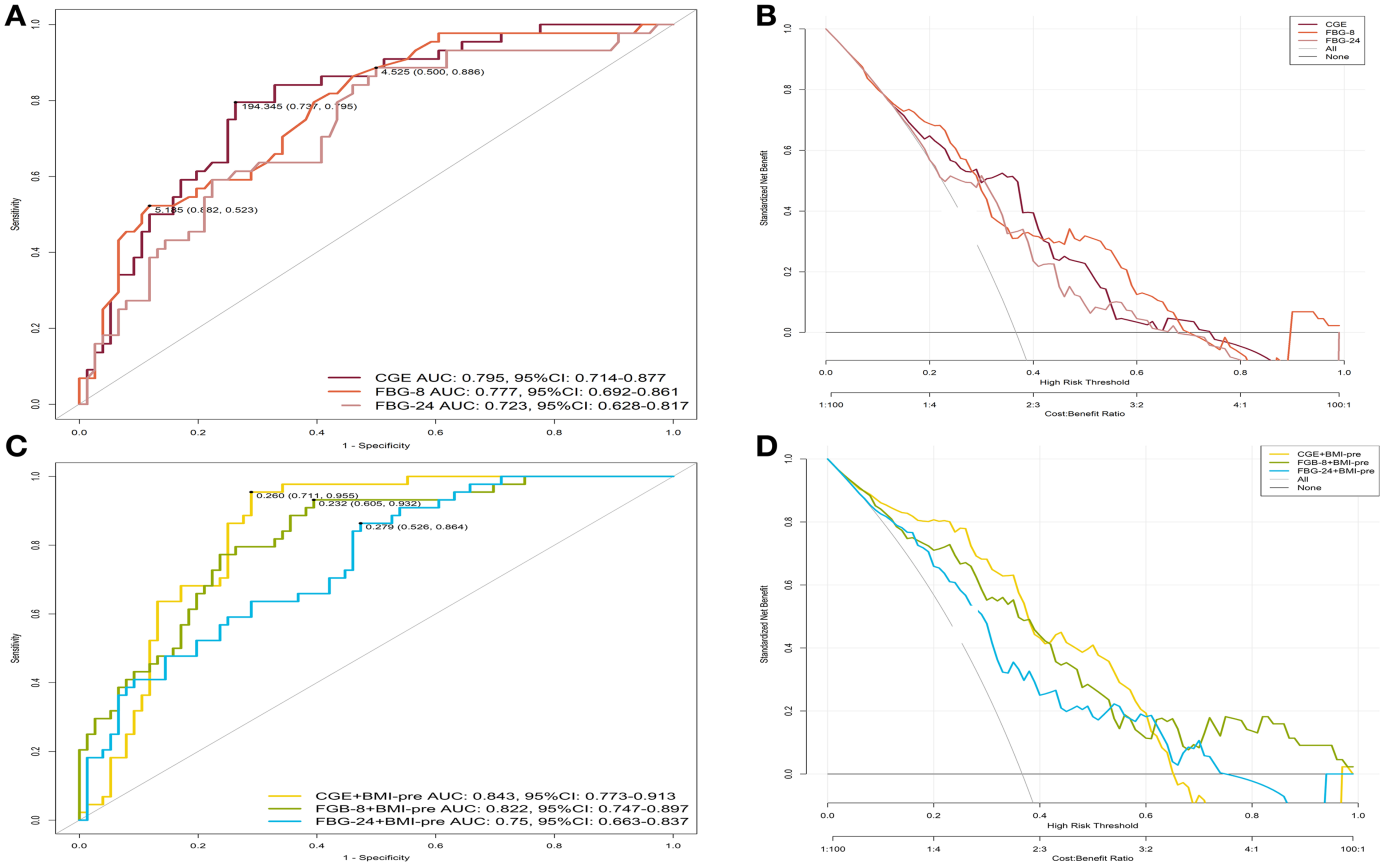
Diagnostic efficacy of ROC curves of CGE and FBG combined with BMI-pre for GMA. **(A)** ROC curves of univariate models (CGE, FBG-8, FBG-24). **(B)** DCA curves of univariate models. **(C)** ROC curves of models incorporating BMI-pre. **(D)** DCA curves of models incorporating BMI-pre. Abbreviation: AUC, the area under the curve; BMI, body mass index; CGE, cumulative glycemic exposure; CI, confidence interval; FBG, fasting blood glucose; GMA, glucose metabolism anomaly; ROC, Receiver Operating Characteristic.

To evaluate the predictive performance of CGE and trimester-specific FBG measurements for postpartum GMA, ROC curves were constructed, and the AUC, sensitivity, specificity, and optimal cutoff values were calculated (Figure 4). For CGE, the AUC was 0.795 (95% CI: 0.714–0.877), with an optimal cutoff value of 194.35 mmol/L·week, yielding a specificity of 73.7% and a sensitivity of 79.5%. FBG at 8 weeks of gestation had an AUC of 0.777 (95% CI: 0.692–0.861), with a cutoff value of 5.18 mmol/L, a specificity of 88.2%, and a sensitivity of 52.3%, while FBG at 24 weeks showed an AUC of 0.723 (95% CI: 0.628–0.817), with a cutoff value of 4.25 mmol/L, a specificity of 50.0%, and a sensitivity of 88.6%.

Combining FBG with pre-pregnancy BMI improved the AUC to 0.822 (95% CI: 0.747–0.897) at 8 weeks (specificity 60.5%, sensitivity 93.2%) and to 0.750 (95% CI: 0.663–0.837) at 24 weeks (specificity 52.6%, sensitivity 86.4%). The combination of CGE with pre-pregnancy BMI yielded the highest AUC of 0.843 (95% CI: 0.773–0.913), with a specificity of 79.1% and a sensitivity of 95.5%, outperforming each individual predictor. Bootstrap internal validation produced an optimism-corrected AUC of 0.831 (95% CI: 0.760–0.902), supporting model stability. These results suggest that combining CGE with pre-pregnancy BMI may enhance risk identification, although its advantage over early-pregnancy FBG requires further validation.

## 4 Discussion

Based on a Chinese pregnancy cohort, this study is the first to systematically examine the association between CGE during pregnancy and GMA at 4–7 years postpartum and to compare the predictive performance of CGE with that of single FBG measurements across trimesters. In addition to GMA, we also systematically evaluated glycemic variability, IR, and cardiometabolic markers, thereby providing a basis for a comprehensive understanding of the long-term consequences of GDM.

Our results showed that each 1-unit increase in CGE was associated with a 4.0% higher risk of postpartum GMA (aOR = 1.040, 95% CI: 1.01–1.07); the risks in T2 and T3 were 3.85- and 15.82-fold higher than the risks in T1. Consistent with previous studies, a continuous positive association was observed between gestational glucose levels and postpartum dysglycemia (14–17). However, prior investigations have largely relied on single or mean glucose values, which fail to fully capture the cumulative burden of glycemic exposure. By integrating multiple FBG measurements using the area under the curve approach, our study more accurately quantified the long-term glycemic load during pregnancy, thereby providing further support for the “cumulative exposure” hypothesis—that both the duration and intensity of hyperglycemia jointly determine long-term metabolic risk (18). RCS analysis revealed a non-linear CGE–GMA association, with the risk increasing substantially above a CGE of 192.67 mmol/L week. Although this exploratory inflection point may offer a reference for glycemic target setting during pregnancy, it requires independent validation in larger cohorts before any clinical application. The CGE unit (mmol/L × week) quantifies cumulative fasting glucose exposure; for example, a mean FBG of 5.0 mmol/L sustained over 38 weeks would yield a CGE of approximately 190 mmol/L·week, which closely approaches the observed threshold. Similar non-linear patterns were also observed for FBG at 24 and 34 weeks of gestation, whereas FBG at 8 weeks exhibited a linear association. These patterns may reflect distinct pathophysiological mechanisms across trimesters—early-pregnancy FBG primarily reflects basal IR, while mid-pregnancy hyperglycemia and late-pregnancy hyperglycemia are additionally driven by the superimposed effects of placental hormones (15, 19, 20).

In our study, DBP, but not SBP, exhibited significant differences across the CGE tertiles. The relatively young age of our cohort may partially explain the lack of SBP changes, since age-related SBP elevation typically becomes more apparent after the fifth decade. Furthermore, increased arterial stiffness, often observed in hyperglycemic states, influences SBP primarily through decreased aortic compliance, whereas DBP alterations are more dependent on peripheral vascular resistance, which is more sensitive to IR and chronic inflammation. Extended follow-up studies are needed to confirm these observations and elucidate the underlying mechanisms (21).

Furthermore, our study demonstrated that women with higher CGE not only had an elevated incidence of GMA but also exhibited worse glycemic variability metrics at 4–7 years postpartum, as reflected by significantly higher SD, CV, MAGE, LAGE, MODD, and ADRR, whereas TIR did not differ significantly. These findings suggest that, in the early stages of glucose dysregulation, glycemic fluctuations may precede a pronounced increase in mean glucose, a finding consistent with prior reports (22). Increased glycemic variability can provoke oxidative stress and endothelial dysfunction, thereby accelerating atherosclerosis (23, 24). Moreover, women with higher CGE had significantly elevated postpartum diastolic blood pressure, triglycerides, and HOMA-IR, indicating a clustering of cardiovascular predictors (25). These findings support the notion that GDM serves as an independent predictor of cardiovascular disease (26, 27) and underscore the importance of addressing not only glycemic levels but also glycemic variability and multiple cardiovascular predictors in postpartum care.

Subgroup analysis revealed a stronger CGE–GMA association in women with pre-pregnancy overweight/obesity (BMI ≥ 24 kg/m^2^) (OR = 1.11, *p* = 0.005) than in those with normal BMI (*p* = 0.103), with a significant interaction. A positive additive interaction was confirmed (RERI = 1.02, AP = 15.3%, S = 1.21). These findings have clinical relevance: Overweight/obesity is itself a pro-inflammatory, insulin-resistant state, and pregnancy hyperglycemia superimposed on it may synergistically accelerate progression to GMA (28, 29). Mechanistically, adipose-derived free fatty acids and cytokines worsen insulin resistance (IR), while hyperglycemia further impairs *β*-cell function. Thus, even modest glucose elevations in women with pre-pregnancy overweight/obesity warrant more intensive intervention. Nonetheless, owing to the limited subgroup sample sizes and the absence of a significant multiplicative interaction, this additive finding should be interpreted as hypothesis-generating, and external validation is warranted to confirm its generalizability.

In this study, FBG levels at 8 and 24 weeks of gestation were significant predictors of GMA, while FBG at 34 weeks was not. This seemingly paradoxical finding underscores the importance of the “time window” of glycemic exposure (19, 30). A single late-pregnancy FBG can be substantially affected by short-term diet and physical activity (31), and the universally elevated glucose levels in late gestation narrow interindividual variability, reducing predictive utility. In contrast, CGE integrates information across the entire pregnancy, offering greater stability and reliability. ROC analysis further confirmed that CGE (AUC = 0.795) outperformed any single FBG, and the addition of pre-pregnancy BMI increased the AUC to 0.843 (sensitivity: 85.5%, specificity: 79.1%). These results suggest that, in clinical practice, CGE derived from multiple FBG measurements, combined with pre-pregnancy BMI, can help identify women at high risk for postpartum GMA.

This study offers the following implications for the postpartum management of women with GDM. First, glycemic control during pregnancy should emphasize sustained, whole-course management rather than reliance on a single fasting glucose measurement. Second, the combination of CGE and pre-pregnancy BMI can serve as a simple and effective risk stratification tool, with an optimal cutoff value of 194.35 mmol/L·week for CGE; this threshold can be used clinically to identify high-risk individuals for early lifestyle intervention. Third, for women with pre-pregnancy overweight or obesity, intensified postpartum glucose monitoring is warranted even when CGE falls below the threshold, given their higher baseline risk and pronounced additive effects.

This study has several limitations. First, the small, single-center sample limits the generalizability of the findings. Second, CGE was calculated from only three fasting glucose measurements, potentially underestimating cumulative exposure; future studies incorporating CGM would provide more precise metrics. Third, the 4–7-year follow-up captures intermediate-term rather than long-term outcomes, and longer surveillance is needed. Fourth, we acknowledge that unmeasured or incompletely adjusted confounders (e.g., breastfeeding, lifestyle, and family history) may have contributed to residual confounding. As these variables were not systematically collected in the original cohort, their potential impact warrants further investigation. Additionally, only 15% of participants were aged ≥35 years, limiting the statistical power of age-stratified analyses. Future multicenter, large-scale studies are warranted to validate CGE cutoff thresholds, evaluate whether CGE-guided interventions (e.g., intensive lifestyle modification and metformin) can reduce long-term T2DM risk, and integrate postpartum CGM-based variables to refine risk prediction models.

## 5 Conclusion

This cohort study identifies elevated pregnancy CGE as an independent predictor of postpartum GMA. The combined model with pre-pregnancy BMI further improved prediction (AUC = 0.843). A non-linear dose–response relationship emerged, with a threshold at 192.67 mmol/L·week, and the association was more pronounced in women with pre-pregnancy overweight/obesity (BMI ≥ 24 kg/m^2^). These results underscore the value of comprehensive gestational glycemic management and offer a quantitative tool for long-term risk stratification. Future multicenter studies should validate generalizability and explore whether CGE-guided strategies reduce long-term risks of T2DM and cardiovascular disease.

## Data Availability

The raw data supporting the conclusions of this article will be made available by the authors, without undue reservation.
